# Case Report: Concomitant coronary stent and femoral artery thrombosis in the setting of heparin-induced thrombocytopenia

**DOI:** 10.12688/f1000research.19041.1

**Published:** 2019-05-15

**Authors:** Mejdi Ben Messaoud, Mezri Maatouk, Mohamed Mehdi Boussaada, Marouane Mahjoub, Walid Mnari, Habib Gamra

**Affiliations:** 1 Cardiology A Department, Fattouma Bourguiba University Hospital, Monastir, Monastir, Monastir, 5000, Tunisia; 2 Radiology Department, Fattouma Bourguiba University Hospital, Monastir, Monastir, Monastir, 5000, Tunisia

**Keywords:** Key words: heparin; thrombocytopenia; thrombosis; coronary artery; stent; myocardial infarction; femoral artery; coronary angioplasty; thrombectomy

## Abstract

Heparin induced thrombocytopenia (HIT) is a rare but potentially life threatening  adverse drug reaction. We report an unusual case of concomitant subacute coronary stent and femoral artery thrombosis secondary to HIT. In the current era of extensive growth of heparin use and percutaneous coronary interventions, it’s important for clinicians to remember that such complication might occur and should be prevented.

## Introduction

Heparin is a commonly used anticoagulant for hospitalized patients, but its use can lead to devastating complications, such as heparin-induced thrombocytopenia (HIT) ^1^. We report an unusual case of concomitant subacute coronary stent and femoral artery thrombosis in the setting of HIT. This case highlights the importance of considering HIT as a cause of coronary or arterial thrombosis few days after heparin exposure.

## Case report

A 66-year-old male patient, with a history of smoking (30 pack-years) and no known medical or surgical history, was admitted in our department for a spontaneously resolved inferior ST elevation myocardial infarction (STEMI). The intra-hospital treatment included enoxaparin 0.6 ml twice a day, clopidogrel 75 mg once a day, aspirin 100 mg once a day, bisoprolol 2.5 mg once a day and atorvastatin 40 mg once a day. The coronary angiogram (performed at day 3 through the right radial artery) showed a severe thrombotic lesion of the distal circumflex. The patient underwent an ad-hoc successful angioplasty of the circumflex with a drug eluting (everolimus) stent. Initial laboratory tests at admission were normal except elevated troponin. Echocardiography showed a 65% left ventricular ejection fraction. The patient was discharged after 5 days of anticoagulation by low molecular weight heparin (enoxaparin). Laboratory tests were not controlled during the hospitalization. The discharge treatment included clopidogrel 75 mg once a day, aspirin 100 mg once a day, bisoprolol 2.5 mg once a day and atorvastatin 40 mg once a day.

**Figure 1.**
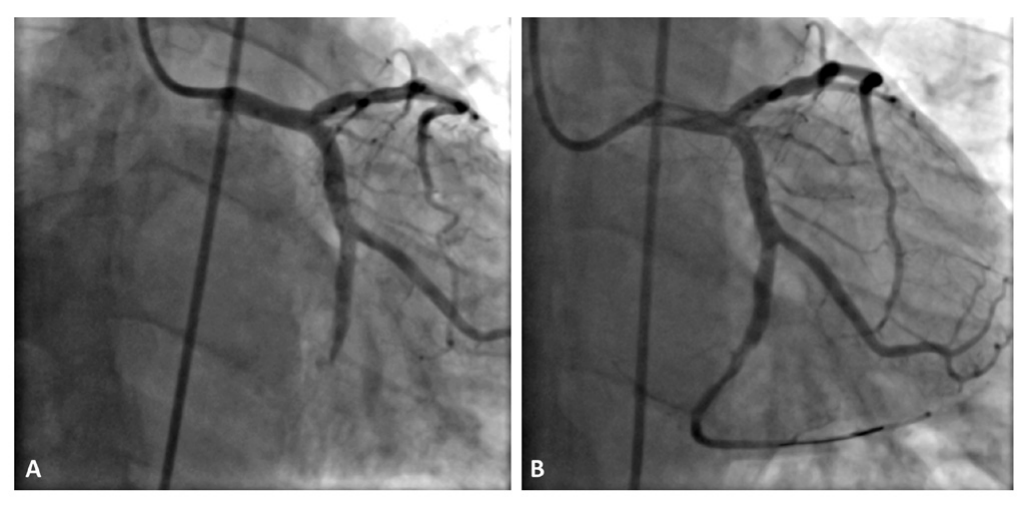
Coronary angiogram images. ( **A**) The intra-stent thrombosis of the circumflex coronary artery. ( **B**) The final result after successful angioplasty of the circumflex coronary artery.

**Figure 2.**
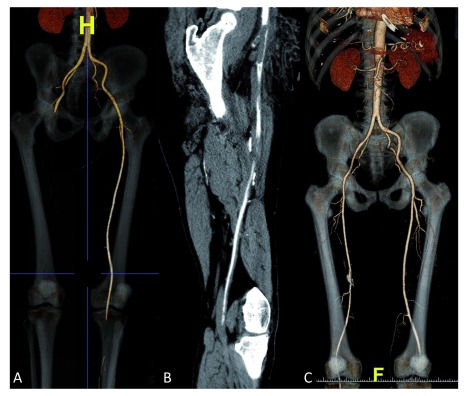
Lower limb contrast enhanced computed tomography images. ( **A**, **B**) show the thrombotic occlusion of the right common femoral artery. ( **C**) Reperfusion of the right femoral artery after thrombectomy.

One week later, the patient was referred again to our department for both chest and right lower limb pain. The electrocardiogram showed an inferior STEMI and the physical exam of the right lower limb found ischemic signs with absence of the femoral pulse. There was no history of aspirin or clopidogrel discontinuation. An urgent coronary angiogram (performed through the left femoral artery) showed total thrombosis of the circumflex stent ( Figure 1A). The patient underwent a successful primary angioplasty of the circumflex by simple balloon ( Figure 1B). Urgent lower limb contrast-enhanced computed tomography was performed immediately after the angioplasty, revealing total acute thrombosis of the right common femoral artery ( Figure 2A, B). The patient underwent an urgent successful thrombectomy with Fogarty catheter. Immediate evolution was favorable with total regression of coronary and right lower limb ischemic signs. Laboratory tests showed a marked fall in the platelet count (68,000/L) which was normal (364,000/L) in the previous hospitalization. A diagnosis of concomitant coronary stent and femoral artery thrombosis due to HIT was strongly suspected (4T score = 8). Our therapeutic strategy was immediate discontinuation of low molecular weight heparin (enoxaparin), aspirin and clopidogrel with strict daily control of platelet count. During this period, no alternative anticoagulation was initiated because of the unavailability of direct thrombin inhibitors in our center. Anticoagulation with a vitamin K antagonist (acenocoumarol 4 mg once a day) and dual antiplatelet therapy with aspirin 100 mg once a day and clopidogrel 75 mg once a day were initiated at day 3 once platelet count had recovered. The in-hospital outcome was favorable and the patient was discharged after 15 days on acenocoumarol 4 mg once a day, aspirin 100 mg once a day and clopidogrel 75 mg once a day. The 3-month follow-up, with controlled blood tests and lower limb contrast-enhanced computed tomography showing total reperfusion of the right femoral artery ( Figure 2C), was unremarkable.

## Discussion

HIT is defined as a sudden fall in the platelet count (e.g. <100,000 per L or >50% drop from baseline) a few days after heparin use. Its incidence ranges from <1% to 7% depending on the heparin type (more than 10 times higher with unfractionated heparin compared to low-molecular-weight heparin), duration of heparin exposure and patient population ^1^. There are two types of HIT, with type I HIT the most common form. Type 1 HIT is due to the direct effect of heparin on platelets and may manifest as only a slight decrease in platelet count, mostly within 2 days of commencing heparin. Type II HIT is secondary to the formation of antibodies against the heparin-platelet factor 4 complex, resulting in 50% of cases in thrombotic events, mostly in veins, within 5 to 14 days. Coronary artery thrombosis secondary to HIT is very rare and usually occurs in the setting of coronary stents or bypass grafts ^2^. However, the concomitant occurrence of coronary stent and other arterial site thrombosis secondary to HIT is very rare and few cases have been reported in the literature ^3^. The use of scoring systems such as the “4T score” is helpful in assessing the pretest probability of HIT ^4^. Platelet factor 4–heparin antibody tests should be ordered only if the diagnosis of HIT is strongly suggested by clinical features ^5^.

Treatment of HIT requires immediate discontinuation of all heparin products and initiation of alternative therapeutic dose anticoagulation, including direct thrombin inhibitors (argotraban, bivalirudin, fondaparinux, danaparoid) or direct oral anticoagulants (apixaban or rivaroxaban or dabigatran) ^6^. The decision to continue antiplatelet therapy during treatment with a non-heparin anticoagulant may be influenced by the risk of vascular events and bleeding. Routine platelet transfusion is not recommended for patients with acute HIT and thrombosis or average bleeding risk, but it may be an option for patients with active bleeding or at high bleeding risk. In the acute phase of HIT with life-threatening thrombosis, bivalirudin (or argatroban if bivalirudin is unavailable) is the best option for alternative anticoagulation therapy ^7^. Primary angioplasty and thrombectomy with Fogarty catheter should be recommended in the setting of life-threatening thrombosis with STEMI or acute limb thromboembolism ^8^. Oral anticoagulation is required for at least 3 months, preferably with direct oral anticoagulant, which can be initiated at the first day. Warfarin is the most highly recommended vitamin K antagonist when indicated, and should not be given until platelets have substantially recovered (e.g. usually to at least 150 000 per L) ^7^. In our case, acenocoumarol was the sole available alternative anticoagulation therapy. It was initiated at day 3 after platelet count recovery and was continued for 3 months.

## Conclusion

Concomitant coronary and femoral artery thrombosis due to HIT is a rare life-threatening complication of heparin therapy. The present case highlights the importance of considering such diagnosis among patients with prior heparin exposure. Prompt identification and management of this disorder is critically important to avoid devastating complications. To prevent such events, strict control of platelet count during heparin therapy is of paramount importance.

## Data availability

All data underlying the results are available as part of the article and no additional source data are required.

## Consent

Written informed consent for publication of their clinical details was obtained from the patient.
