# Supplementary material for: Case Report: Concomitant coronary stent and femoral artery thrombosis in the setting of heparin-induced thrombocytopenia
Source: F1000Res. 2019 May 15;8:667. [Version 1] doi: 10.12688/f1000research.19041.1 (PMC6611129; doi:10.12688/f1000research.19041.1)
Supplement: Peer review history

# Peer review: Reviewer response for version 1

**DOI:** 10.5256/f1000research.20868.r49698

**Published:** 2019-07-04

**Authors:** Marouane Boukhris

**Affiliations:** 1 Centre Hospitalier de l'Université de Montréal, Quebec, Canada

In this current manuscript, the authors reported the case of a STEMI patient who suffered from heparin-induced thrombocytopenia resulting not only in stent thrombosis but also in femoral artery thrombotic occlusion. Overall, the paper is well written and of interest for the reader. It is suitable for indexing in its actual version.

I confirm that I have read this submission and believe that I have an appropriate level of expertise to confirm that it is of an acceptable scientific standard.

# Peer review: Reviewer response for version 1

**DOI:** 10.5256/f1000research.20868.r48571

**Published:** 2019-06-04

**Authors:** Batric Popovic

**Affiliations:** 1 Department of Cardiology, Centre Hospitalier Universitaire Brabois, Nancy, France

**Comments:**

 I read with great interest the report written by Mejdi Ben Messaoud et al. about an unusual case of concomitant subacute coronary stent and femoral artery thrombosis secondary to heparin induced thrombocytopenia. There had been previous reported cases of HIT causing coronary thrombus formation but not with concomitant extracoronary thrombotic events.

 The authors confirm that a heparin therapy needs a platelet count at least 2/ week which is a well-known recommendation. I think that authors should also emphasize that a prolonged and unnecessary anticoagulation after percutaneous coronary intervention may be deleterious. Indeed, different studies reported that in elective PCI or after primary angioplasty after myocardial infarction, the anti coagulation should be early discontinued.

 This report needs several minor modifications as follows:

**Abstract:**

“In the current era of extensive growth of heparin use and percutaneous coronary interventions, it’s important for clinicians to remember that such complication might occur and should be prevented.”

 This sentence should be modified: PCI required perprocedural anticoagulation but dose and duration of anticoagulation have been decreased last decade. (See: Montalescot STEEPLE investigators and Futura/OASIS 8).

**Case report:**

“A 66-year-old male patient, with a history of smoking (30 pack-years) and no known medical or surgical history:”.

 30 pack years should be deleted and “no known”: means without?

Echocardiography showed a 65% left ventricular ejection fraction.

 The authors should specify the presence or not of an intra cardiac mass. Indeed, one of the most frequent underlying cardiac diseases concomitant coronary and extra coronary acute thrombotic events is intracardiac mass/tumor.

The in-hospital outcome was favorable and the patient was discharged after 15 days on acenocoumarol 4 mg once a day, aspirin 100 mg once a day and clopidogrel 75 mg once a day.

 Indicate the duration of this antithrombotic strategy in this paragraph and not in the discussion.

“The 3-month follow-up, with controlled blood tests and lower limb contrast-enhanced computed tomography showing total reperfusion of the right femoral artery ,was unremarkable.”

 The sentence may be modify: “At 3 month follow up, no recurrent thrombotic events occurred, controlled blood tests were normalized and low limb contrast-enhanced computed tomography completely reperfused."

I confirm that I have read this submission and believe that I have an appropriate level of expertise to confirm that it is of an acceptable scientific standard.
